# M2‐like macrophage‐derived exosomes facilitate metastasis in non‐small‐cell lung cancer by delivering integrin αVβ3

**DOI:** 10.1002/mco2.191

**Published:** 2022-12-23

**Authors:** Lamei Huang, Fang Wang, Xueping Wang, Chaoyue Su, Shaocong Wu, Chuan Yang, Min Luo, Jianye Zhang, Liwu Fu

**Affiliations:** ^1^ State Key Laboratory of Oncology in South China Collaborative Innovation Center for Cancer Medicine Guangdong Esophageal Cancer Institute Sun Yat‐sen University Cancer Center Guangzhou P. R. China; ^2^ Guangzhou Municipal and Guangdong Provincial Key Laboratory of Molecular Target & Clinical Pharmacology NMPA and State Key Laboratory of Respiratory Disease School of Pharmaceutical Sciences and the Fifth Affiliated Hospital Guangzhou Medical University Guangzhou P. R. China

**Keywords:** exosomes, integrin αVβ3, M2‐like macrophage, metastasis, NSCLC

## Abstract

Metastasis is the most prevalent cause of cancer deaths, and immunological components of the tumor microenvironment, especially tumor‐associated macrophages (TAMs), play a vital role in cancer metastasis. However, the underlying mechanisms of TAMs on non‐small‐cell lung cancer (NSCLC) metastasis remain largely unexplored. Herein, we demonstrated that M2‐like TAMs facilitate the migration and invasion of cancer cells in vitro and in vivo through intercellular delivery of M2‐like macrophage‐derived exosomes (M2‐exos). Importantly, we found that M2‐exos had considerably higher levels of integrin (ITG) αV and β3. The impact of M2‐like macrophage‐mediated invasion and migration of NSCLC cells was clearly decreased when ITG αVβ3 was blocked. Mechanistically, exosomal ITG αVβ3 produced from M2‐like macrophages successfully triggered the focal adhesion kinase signaling pathway in recipient cells, boosting the migratory and invasive abilities of NSCLC cells. Clinically, we found that metastatic NSCLC patients had greater ITG αV and β3 expression, which was associated with a worse prognosis. This study reveals a novel mechanism by which M2‐exos significantly increased NSCLC cell migration and invasion by delivering integrin αVβ3. Exosomal ITG αVβ3 can be used as a potential prognostic marker, and blocking ITG αVβ3 could be a viable treatment option for preventing tumor metastasis.

## Graphical abstract

Tumor‐associated macrophage‐derived exosomes were significantly rich in integrin (ITG) αV and β3. Exosomes derived from tumor‐associated macrophages are taken up by tumor cells and activate the FAK signaling pathway to enhance the migration and invasion ability of tumor cells.

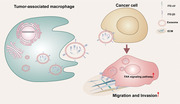

## 1 INTRODUCTION

Globally, lung cancer is the dominant forerunner of cancer‐related fatalities, with non‐small‐cell lung cancer (NSCLC) accounting for 80%–85% of deaths.^1^, ^2^ Metastasis occurs frequently in patients with NSCLC, among which brain metastases and bone metastases are the most common.^3^ The 5‐year survival rate of patients with metastasis is less than 15%.^3^ Multiple gene mutations related to NSCLC metastasis have been identified, including mutations in EGFR, VEGF, KRAS, p53, and PTEN.^4^, ^5^, ^6^ However, with the improvement of the understanding of various aspects of tumors, increasing evidence shows that the tumor microenvironment (TME) has a crucial role in metastasis, whether it is primary site invasion or distant metastatic colonization.^7^, ^8^, ^9^, ^10^

The confrontation between tumor cells and immune cells determines the initiation and progression of tumors In the TME.^11^ Although adaptive immunity is widely regarded as the main force against tumors, increasing evidence suggests that innate immune cells, especially tumor‐associated macrophages (TAMs), also play an important role in this battle.^12^, ^13^, ^14^ Tumor cells recruit and civilize macrophages in the TME to differentiate into TAMs by secreting various cytokines, such as CSF1 and CCL2.^15^, ^16^ Generally, TAMs are similar to M2 macrophages in phenotype and function.^17^ TAMs can release various cytokines, such as TGF‐β and EGF, which not only promote tumor proliferation and transformation but also facilitate the establishment of a tumor tolerance microenvironment.^18^, ^19^, ^20^ TAMs can also secrete a series of inflammatory inhibitory molecules, including IL‐10 and IL‐13, which can directly inhibit CD8+ T and CD4+ T‐cell‐mediated tumor killing.^21^, ^22^ TAM infiltration in the TME is linked to poor prognosis in breast, oral, ovarian and bladder cancers, and Hodgkin's lymphoma.^23^, ^24^, ^25^ However, specific evidence linking TAMs and NSCLC is still lacking, particularly in terms of NSCLC metastasis.

Extracellular vesicles (EVs) carry bioactive molecules that affect the extracellular environment and the immune system.^26^ According to the MISEV2018, EV is recommended for the use of operational terms for EV subtypes that refer to physical characteristics, biochemical composition, and descriptions of conditions or cell of origin.^27^ The primary EV class includes apoptotic bodies (800–5000 nm), microvesicles (200–1000 nm), and exosomes (30–200 nm). Exosomes are lipid bilayer membrane vesicles originating from endocytosis that are key mediators of intercellular cross talk.^28^ Exosomes contain a variety of bioactive molecules, including proteins, lipids, RNAs, and DNAs, which can be transferred to recipient cells and mediate their biological functions.^29^ Accumulating research has shown that tumor‐derived exosomes are associated with tumor growth, drug resistance, metastasis, and remodeling of the tumor immune microenvironment.^30^, ^31^ Exosomes from lung cancer, for instance, contribute to the polarization of macrophages toward an M2‐like phenotype.^32^ M2 macrophage‐derived exosomes have been found to promote tumor progression and metastasis in colorectal cancer and liver cancer.^33^, ^34^ However, there has been little investigation into the effect of M2‐like macrophage‐derived exosomes (M2‐exos) in metastatic NSCLC.

In this research, we demonstrated that M2‐exos were responsible for NSCLC progression and metastasis both in vitro and in vivo. Importantly, ITG αVβ3 was found to be highly enriched in M2‐exos and was closely associated with NSCLC metastasis. The underlying mechanisms could be that M2‐like macrophage‐derived exosomes mediate ITG αVβ3 transmission to NSCLC cells, which triggers the focal adhesion kinase (FAK) signaling in recipient cells, thus enhancing NSCLC cell migration and invasion. Our findings shed new light on the role of macrophages in tumor metastasis, suggesting that M2‐like macrophage‐derived exosomes play an important role in tumor progression and may become a new target for tumor therapy.

## 2 RESULTS

### 2.1 M2‐like macrophage infiltration is associated with poor clinical outcomes

**FIGURE 1:**
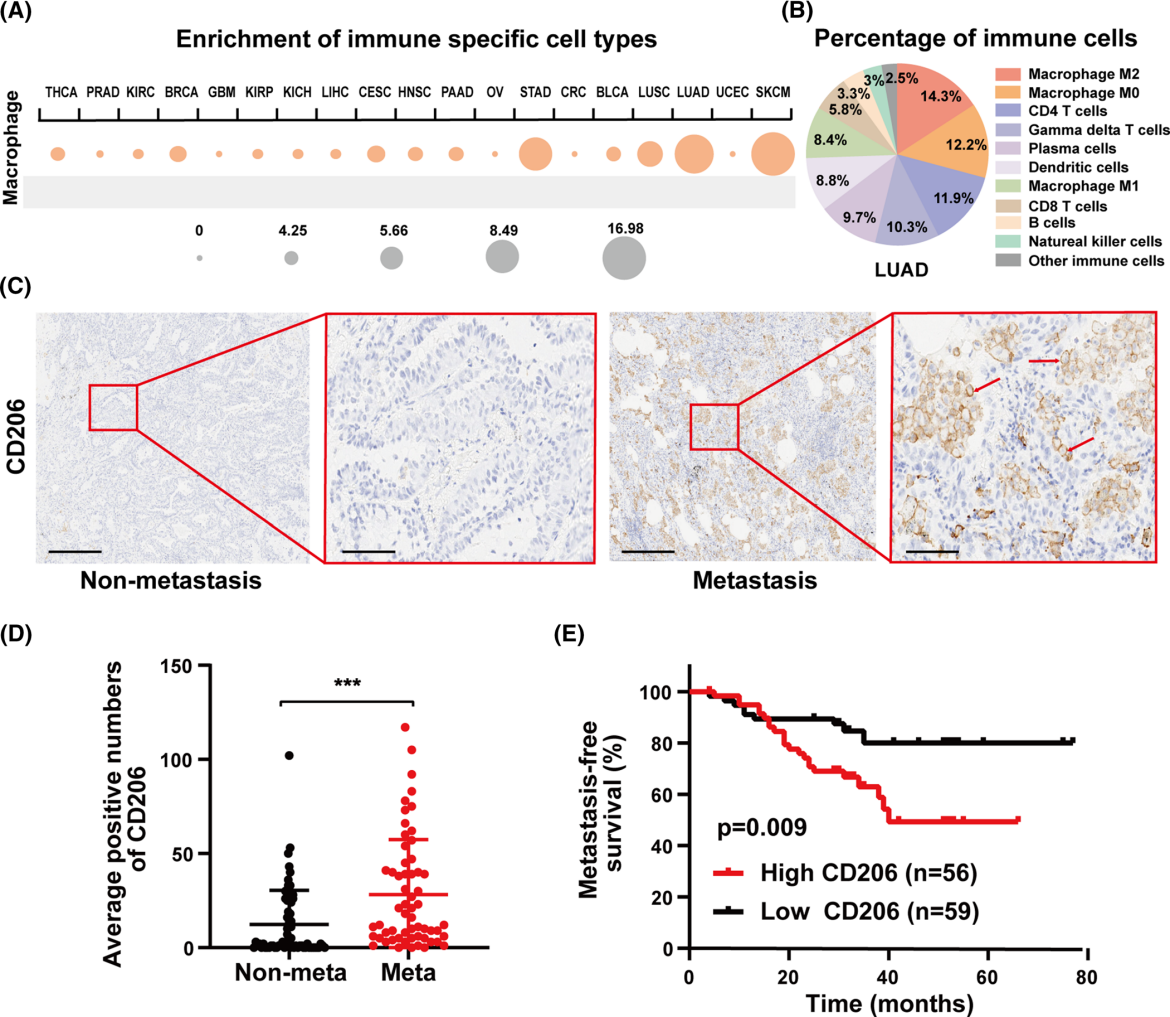
M2‐like macrophage infiltration is associated with poor clinical outcomes. (A) Macrophage enrichment in 19 different solid tumors. The ssGSEA results are shown in a circle plot, with the size of the circles denoting the percentage of patients with NES >0 and *q* value (FDR) <0.1. (B) The average fraction of immunological subpopulations in lung adenocarcinoma (LUAD) specimens (*n* = 574). (C and D) Representative images and statistical results of CD206 immunohistochemical staining in LUAD specimens. The tumor specimens collected from patients were divided into a metastatic group (*n* = 59) and nonmetastatic group (*n* = 67) according to pathological information and follow‐up information. The inclusion criteria for the metastasis group were those with pathological findings of metastases, including distant and lymph node metastases, or patients with metastases after surgery. The red arrow refers to tumor cells with high expression of CD206. Left scale bar, 400 μm; right scale bar, 100 μm. (E) The relationship between CD206 expression and metastasis‐free survival in LUAD patients. There were 59 cases of low CD206 expression and 56 cases of high CD206 expression. Data are shown as the mean ± SEM. BLCA, bladder urothelial carcinoma; BRCA, breast invasive carcinoma; CESC, cervical squamous cell carcinoma and endocervical adenocarcinoma; CRC, colorectal cancer; GBM, glioblastoma multiforme; HNSC, head‐and‐neck squamous cell carcinoma; KICH, kidney chromophobe; KIRC, kidney renal clear cell carcinoma; KIRP, kidney renal papillary cell carcinoma; LIHC, liver hepatocellular carcinoma; LUSC, lung squamous cell carcinoma; meta, metastatic NSCLC; Nonmeta, nonmetastatic NSCLC; NSCLC, non‐small‐cell lung cancer; OV, ovarian serous cystadenocarcinoma; PAAD, pancreatic adenocarcinoma; PRAD, prostate adenocarcinoma; SKCM, skin cutaneous melanoma; STAD, stomach adenocarcinoma; THCA, thyroid carcinoma; UCEC, uterine corpus endometrial carcinoma. ***p < 0.001

To examine the relationship between macrophages and lung cancer, the Cancer Immunome Atlas (https://tcia.at/), an online database, was used to assess macrophage distribution in the TME. According to an analysis of macrophage distribution in various cancers, macrophages were highly enriched in lung adenocarcinoma (LUAD) samples (Figure 1A). In LUAD, M2‐like macrophages made up the largest fraction of immune cells (Figure 1B). TAMs are distinguished by specific surface molecules, such as the mannose receptor CD206, which related to angiogenesis and cancer metastasis.^35^ LUAD specimens were collected to better understand the distribution of M2‐like macrophages in LUAD. M2‐like macrophages were found to be more prevalent in metastatic LUAD specimens (*n* = 59) than in nonmetastatic LUAD specimens (*n* = 67) (Figure 1C,D). Furthermore, high M2‐like macrophage infiltration was linked to a poor prognosis (Figure 1E). Overall, macrophages are the most common immune subgroup in LUAD, and LUAD with high M2‐like macrophage infiltration is more prone to metastasis.

### 2.2 M2‐like macrophages enhance NSCLC cell migration and invasion

**FIGURE 2:**
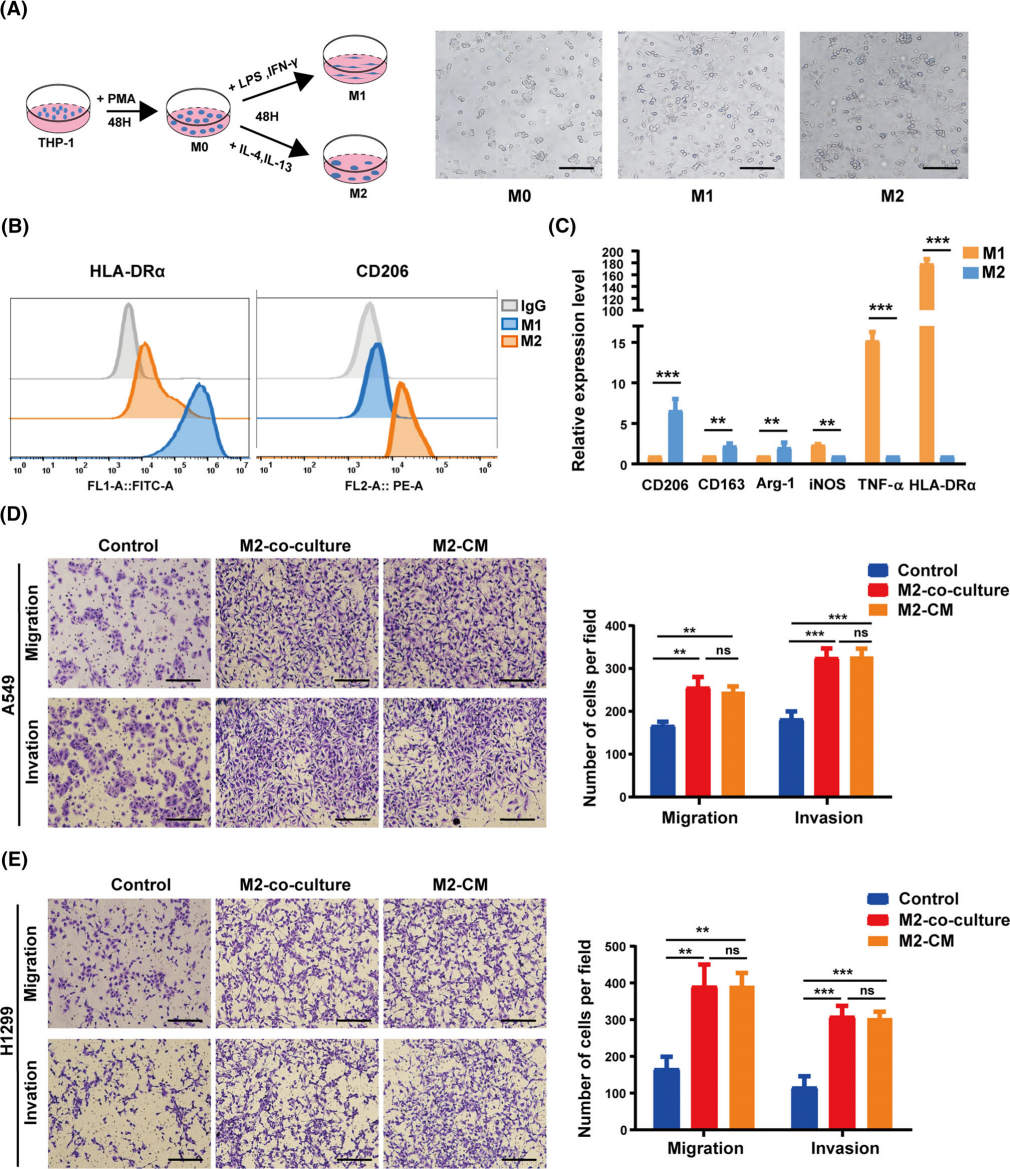
M2‐like macrophages enhance non‐small‐cell lung cancer (NSCLC) cell migration and invasion. (A) Schematic diagram of induced M1‐ or M2‐like macrophages from THP‐1 monocytes. Scale bar, 200 μm. (B) HLA‐DRα/CD206 flow cytometry markers were used to identify M1‐ or M2‐like macrophages. (C) The gene expression levels of M1‐ and M2‐like macrophages were validated by real‐time PCR assays. (D and E) The migration and invasion capacity of cancer cells were determined using Transwell assays after A549 and H1299 cells were cocultured with M2‐like macrophages or pretreated with conditioned media from M2‐like macrophages for 24 h. The left panel shows representative images, whereas the right panel shows migrating cell counts. Scale bar, 150 μm. The previous experiments were repeated at least three times to ensure the accuracy of the data. All data are presented as the means ± SEMs. Arg‐1, arginase 1; HLA‐DRα, major histocompatibility complex, class II, DR alpha; iNOS, inducible nitric oxide synthase; PMA, phorbol‐12‐myristate‐13 acetate; TNF‐α, tumor necrosis factor‐α. *p < 0.05, **p < 0.01, ***p < 0.001

It has been reported that TAMs extensively regulate the tumor progression and metastasis of a variety of tumors.^36^ Typically, TAMs exhibit an M2‐like phenotype. To explore the impact of TAMs on LUAD in vitro, we first successfully constructed an M1/M2‐like macrophage model in vitro using THP‐1 monocytes (Figure 2A). In contrast to M1‐like macrophages, M2‐like macrophages showed increased expression of CD206, CD163, and Arg‐1 (M2 macrophage‐associated marker) along with diminished levels of HLA‐DRα, TNF‐α, and iNOS (M1 macrophage‐associated marker), which was confirmed by flow cytometry and qPCR assays (Figure 2B,C). The induced M2‐like macrophages were then cocultured with NSCLC cells for 24 h, or NSCLC was pretreated with an M2‐conditioned medium. We found that both treatments markedly enhanced the migration and invasion abilities of H1299 and A549 cells (Figure 2D,E). Therefore, we demonstrated in vitro that M2‐like macrophages assist the progression and metastasis of NSCLC.

### 2.3 M2‐like macrophage‐derived exosomes enhance NSCLC cell migration and invasion

**FIGURE 3:**
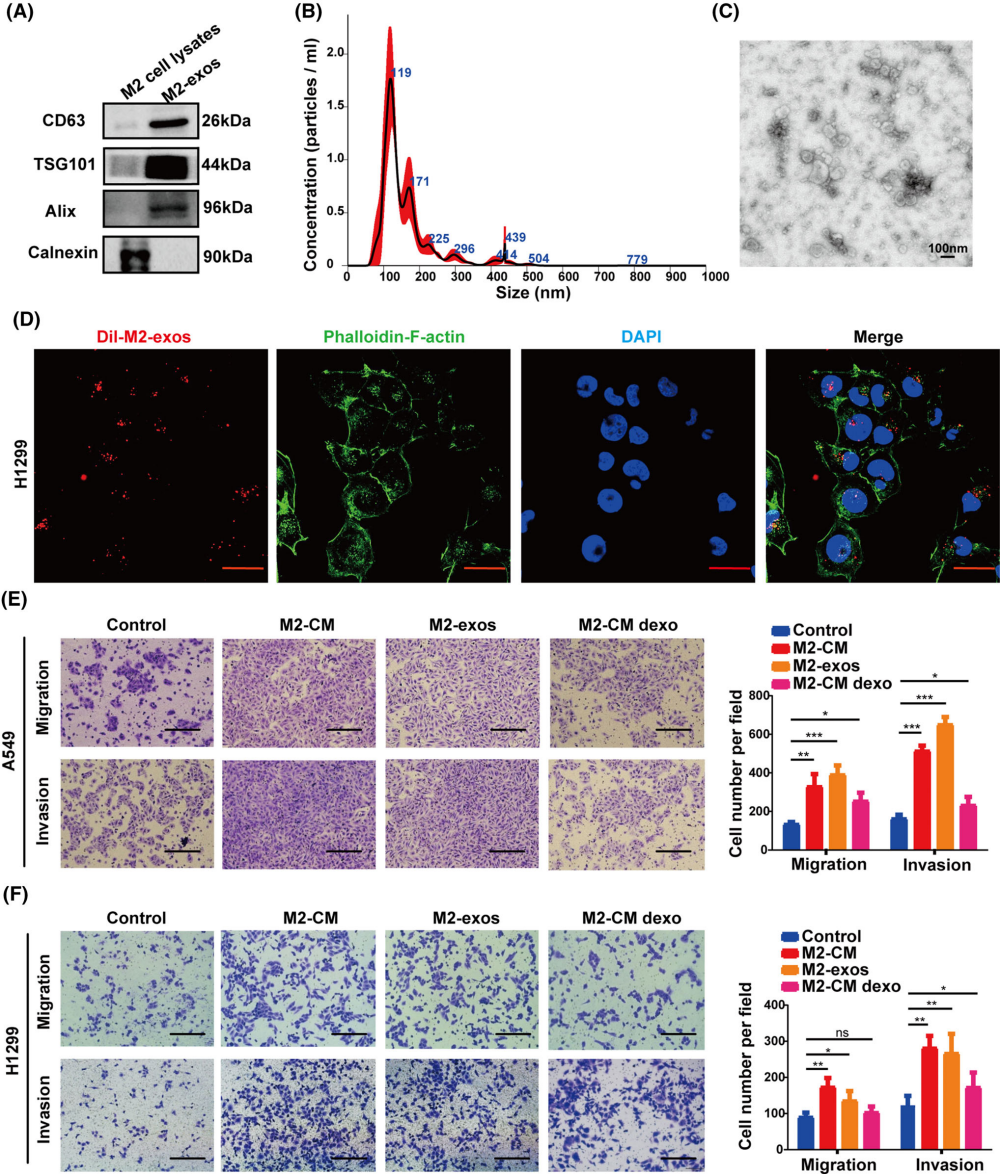
M2‐like macrophage‐derived exosomes enhance non‐small‐cell lung cancer (NSCLC) cell migration and invasion. (A) Western blot of exosomal markers in M2‐like macrophage whole‐cell lysates and M2‐like macrophage‐derived exosomes (M2‐exos). (B) Nanoparticle tracking analysis of M2‐exos. (C) Transmission electron microscope imaging of M2‐exos. (D) The uptake of DiI‐labeled M2‐exos by H1299 cells was detected by confocal microscopy. The cytoskeleton and nucleus of H1299 cells were labeled with FITC‐phalloidin and DAPI, respectively. Scale bar, 20 μm. (E and F) A549 and H1299 cells were pretreated for 24 h with phosphate buffered saline (PBS) (control), M2‐CM, M2‐exos, and M2‐CM dexo, and the capacity of cancer cells to migrate and invade was determined using Transwell assays. The left panel displays representative photos, whereas the right panel displays migrating cell counts. The previous experiments were repeated at least three times to ensure the accuracy of the data. All data are presented as the means ± SEMs. *p < 0.05, **p < 0.01, ***p < 0.001

After pretreatment with M2‐like macrophage‐conditioned medium, we found that migration and invasion of A549 and H1299 cells were significantly increased. Therefore, we wondered if substances derived from M2‐like macrophages were responsible for the remarkable effect. Accumulated evidence suggests that exosomes derived from tumor cells or tumor‐associated stromal cells are involved in tumor metastasis.^37^ In this research, we focus on M2‐like macrophage‐derived 30–200 nm EVs known as exosomes, abbreviated M2‐exos. To investigate whether M2‐exos are related to cancer metastasis, we extracted exosomes from M2‐like macrophage culture medium and subsequently treated NSCLC cells in vitro with these exosomes. Exosomes were validated by Western blot analysis using the exosome‐specific markers, TSG101, CD63, and ALIX as well as the negative marker calnexin (Figure 3A). In addition, nanoparticle tracking analysis and transmission electron microscopy were used to quantify particle size and morphology (Figure 3B,C). To determine whether M2‐exos can be internalized by NSCLC cells, we pretreated H1299 cells with PKH26‐labeled exosomes for 12 h. Confocal fluorescence imaging revealed that M2‐exos tagged with PKH26 were highly absorbed by H1299 cells (Figure 3D).

We hypothesize that M2‐exos promote NSCLC cell migratory and invasive capacities. Therefore, H1299 cells and A549 cells were cocultured with M2‐macrophage‐derived medium (M2‐CM), M2‐exos, and M2‐CM depleted exosomes (M2‐CM dexo), respectively. Transwell assays were employed to assess cancer cell migration and invasion ability. As expected, M2‐exos notably increased the migration and invasion of NSCLC cells but had little impact on cell proliferation (Figures 3E,F and S1). Collectively, our results revealed that M2‐like macrophages enhanced the mobility and aggressiveness of NSCLC cells, which were predominantly dependent on exosomes.

### 2.4 Exosomal ITG αVβ3 derived from M2‐like macrophages is a key player in mediating NSCLC metastasis

**FIGURE 4:**
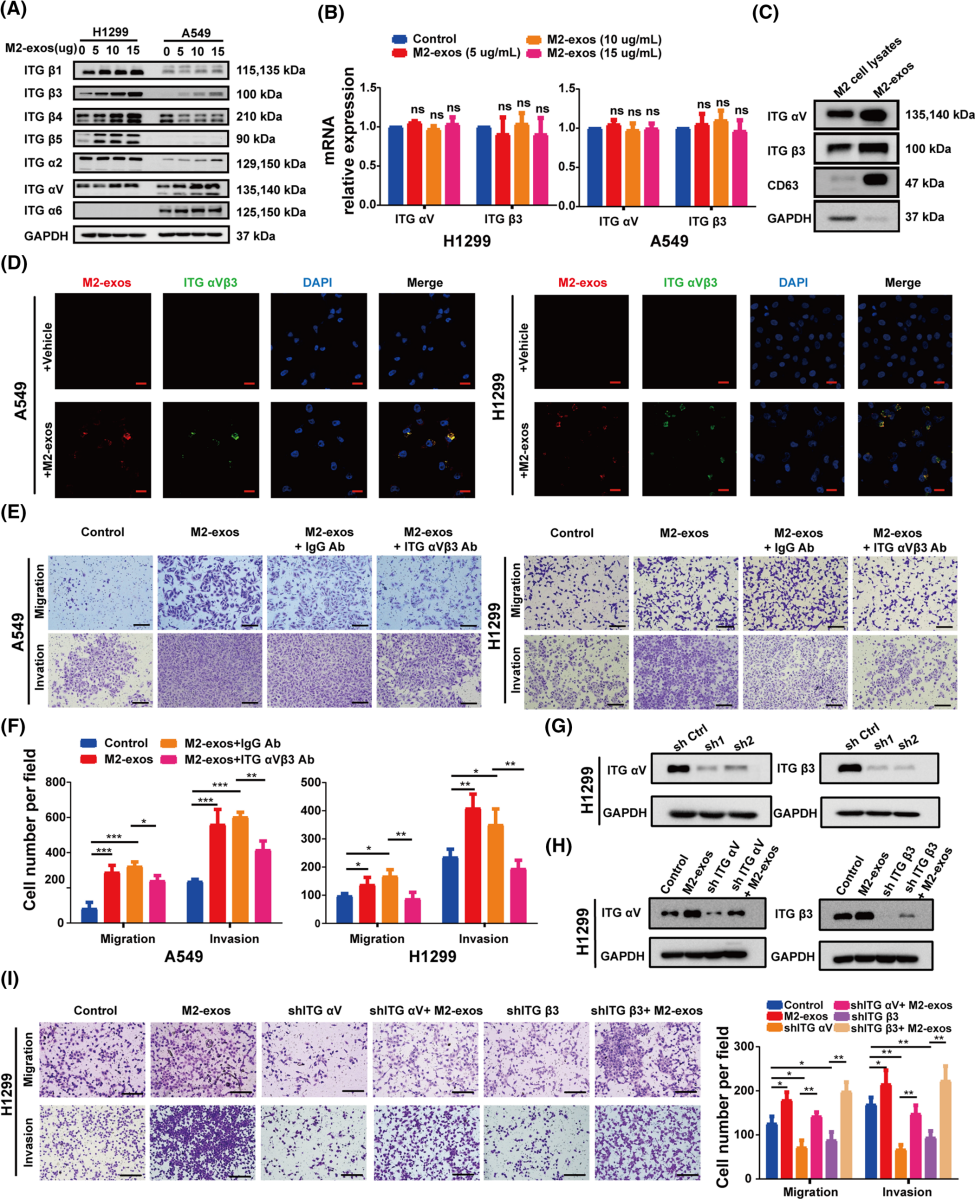
ITG αVβ3 is abundant in M2‐like macrophage‐derived exosomes (M2‐exos) and plays an important role in non‐small‐cell lung cancer (NSCLC) metastasis. (A) After treatment with different concentrations of M2 exos, Western blot analysis of ITG β1, β3, β4, β5, ITG α2, αV, and α6 protein levels in A549 and H1299 cells was performed. (B) Real‐time PCR assays were used to examine the mRNA expression levels of ITG αV and β3 in H1299 cells after treatment with various concentrations of M2‐exos. (C) Western blot of ITG αVβ3 protein levels in M2‐like macrophage whole‐cell lysates and M2‐exos. (D) Representative images of the internalization of M2‐exos (red) containing ITG αVβ3 (green) in H1299 cells. Scale bar, 20 μm. (E and F) A549 and H1299 cells were pretreated with phosphate buffered saline (PBS) (control), M2‐exos, M2‐exos+anti‐IgG blocking Ab or M2‐exos+anti‐ITG αVβ3 blocking Ab for 24 h, and the migration and invasion capacities of cancer cells were detected using Transwell assays. The left panel shows representative images, whereas the right panel shows migrating cell counts. Scale bar, 150 μm. (G and H) The efficacy of ITG αV/β3 silencing by shRNAs was confirmed by Western blot experiments. (I) Transwell assays evaluated the migration and invasion ability of normal H1299 cells or ITG αV‐ and β3‐silenced counterparts treated with or without M2‐exos. The left panel shows representative images, whereas the right panel shows migrating cell counts. Scale bar, 150 μm. The previous experiments were repeated at least three times to ensure the accuracy of the data. All data are presented as the means ± SEMs. ITG α2, integrin α2; ITG α6, integrin α6; ITG αV, integrin αV; ITG αVβ3 Ab, ITG αVβ3 blocking antibody; ITG β1, integrin β1; ITG β3, integrin β3; ITG β4, integrin β4; ITG β5, integrin β5. *p < 0.05, **p < 0.01, ***p < 0.001

According to the evidence presented before, exosomes released by M2‐like macrophages deliver certain components to NSCLC cells, enhancing cancer cell motility and invasion. Previous research demonstrated that NSCLC cell‐derived exosomes played a key role in mediating tumor metastasis by targeting integrin signaling pathways. Previous research revealed that tumor‐derived exosomes with different integrin expression patterns were taken up by different organ‐specific cells, which in turn contributed to the formation of the pre‐metastatic niche and definitive organotropic metastasis.^38^ Integrins, a heterodimeric transmembrane receptor family capable of regulating intercellular interactions with the extracellular matrix, have a key impact on the regulation of a range of tumor cell behaviors, such as proliferation, adhesion, migration, invasion, and survival. Thus, we assumed that integrin might play a key role in M2‐exos in mediating NSCLC metastasis. We discovered that M2‐exo treatment significantly increased the protein expression levels of ITG αV and β3 in H1299 and A549 cells in a concentration‐dependent manner. However, there was no significant change in the mRNA levels of ITG αVβ3 (Figure 4A,B). Therefore, the increased protein expression of ITG αV and β3 in A549 and H1299 cells is not endogenous. We investigated the expression of ITG αV and β3 in M2‐exos to investigate whether exosomes mediate direct intercellular transmission of ITG αVβ3. Western blot experiments revealed that ITG αV and β3 were considerably more abundant in M2‐exos than in M2 macrophage cell lysates (Figure 4C). Furthermore, the colocalization of ITG αVβ3 and exosomes was observed in A549 and H1299 cells cocultured with M2‐exos, indicating that ITG αVβ3 was transported from M2‐like macrophages to NSCLC cells via exosomes (Figure 4D). In conclusion, these results revealed that M2‐exos enriched ITG αVβ3 meaningfully and could be directly transferred to NSCLC cells.

To ascertain whether M2‐exo‐generated ITG αVβ3 on mediates NSCLC cells metastasis, M2‐exos were preincubated with or without an anti‐ITG αVβ3 blocking antibody (ITG αVβ3 Ab) and subsequently cocultured with NSCLC cells to detect their migration and invasion abilities. In this investigation, an IgG blocking Ab (IgG Ab) was used to evaluate the specificity of ITG αVβ3 Ab. As shown, compared to the M2‐exos+IgG Ab group, the M2‐exos+ITG αVβ3 Ab group effectively prevented the invasion and migration of H1299 and A549 cells (Figure 4E,F). To further confirm the critical role of exosomal ITG αVβ3 derived from M2‐like macrophages, ITG αV and β3 expressions were suppressed in H1299 cells employing two distinct shRNAs, and Western blot assays were used to confirm the knockdown efficacy (Figure 4G). Moreover, the downregulation of ITG αV and β3 protein expression in H1299 cells significantly repressed their capacities of migration and invasion, and M2‐exo treatment reversed this inhibitory effect (Figure 4H,I).

### 2.5 M2‐like macrophage‐derived exosomal ITG αVβ3 promotes NSCLC metastasis in vivo

**FIGURE 5:**
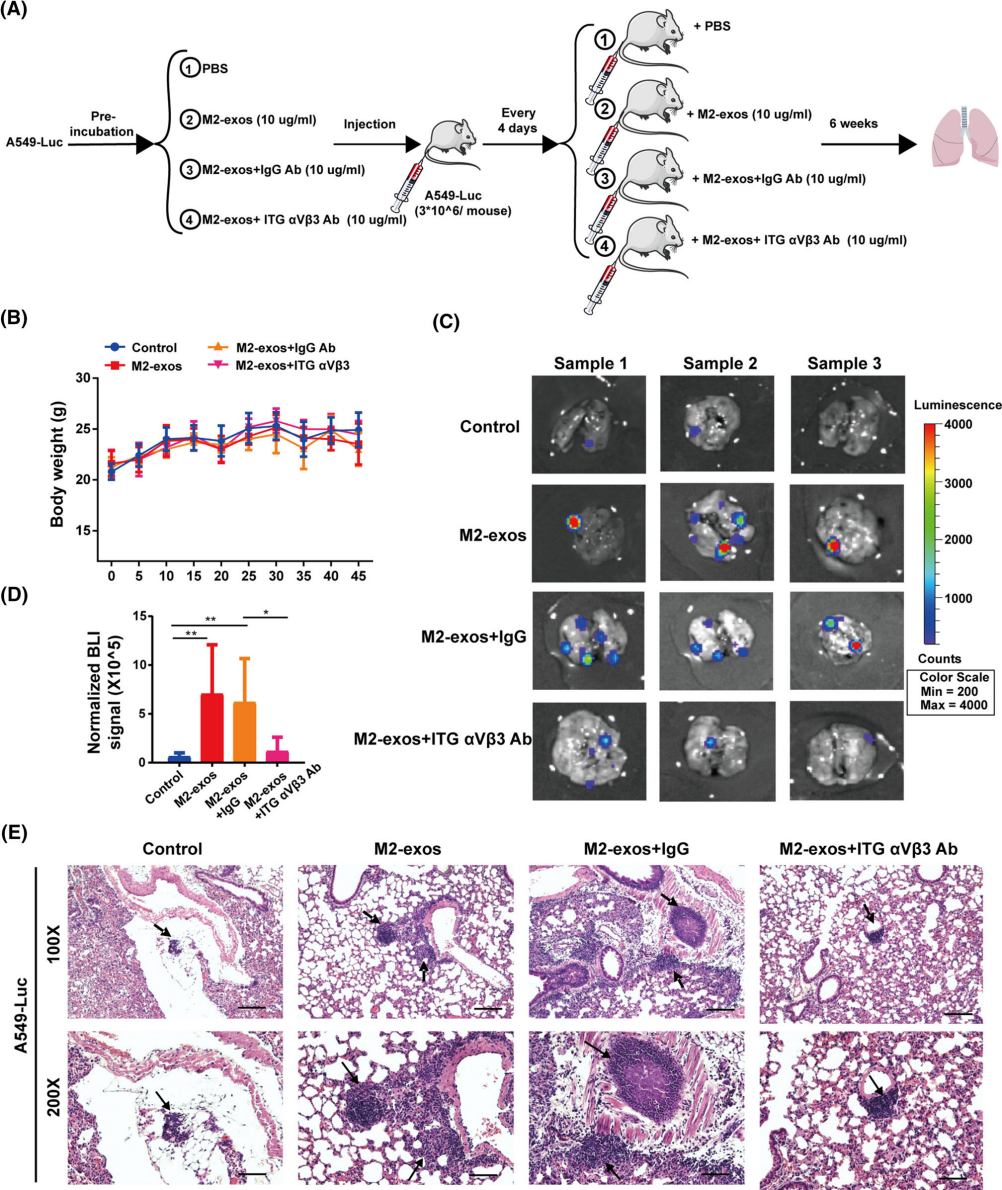
Exosomes secreted from M2‐like macrophages promote non‐small‐cell lung cancer (NSCLC) cell lung metastasis in vivo. (A) Schematic diagram illustrating the experimental process. A549^Luc^ cells were cocultured with phosphate buffered saline (PBS), M2‐like macrophage‐derived exosomes (M2‐exos), M2‐exos+IgG Ab and M2‐exos+ITG αVβ3 Ab, and injected into the caudal veins of male nude mice (six mice for each group). Every 4 days, PBS, M2‐exos, M2‐exos+IgG Ab, and M2‐exos+ ITG αVβ3 Ab were administered into matching mice. (B) A line graph depicting the effects on body weight of mice over the course of an experiment (*n* = 6). (C and D) Representative images and fluorescence intensity statistics of in vitro lung fluorescence imaging of each group. The black arrow refers to typical metastatic tumor lesion in the mouse lung. (E) Hematoxylin and eosin (H&E) staining for A549 cell lung metastases. Top scale bar, 400 μm; bottom scale bar, 200 μm. All data are presented as the means ± SEMs. A549^Luc^, A549 cells expressing luciferase; ITG αVβ3 Ab, ITG αVβ3 blocking antibody. *p < 0.05, **p < 0.01, ***p < 0.001

To investigate whether M2‐exos and its component ITG αVβ3 prime NSCLC lung metastasis in vivo, we constructed A549 cells stably expressing the luciferase gene (A549^luc^), followed by treatment with M2‐exos, M2‐exos+IgG Ab, M2‐exos+ITG αVβ3 Ab and phosphate buffered saline (PBS). Then, A549^luc^ cells with different treatments were injected into the caudal veins of male nude mice, and various treatment interventions were performed as illustrated in the scheme (Figure 5A). There was no notable variation in body mass between the groups throughout the experiment (Figure 5B). When the M2‐exos and M2‐exos+IgG antibody groups were compared to the control group, we discovered a substantial increase in lung metastases. When compared to the M2‐exos and M2‐exos+IgG group, lung metastasis was considerably reduced in the M2‐exos+ITG αVβ3 Ab group (Figure 5C,D). These results suggested that ITG αVβ3 was indeed the main effector molecule mediating M2‐exos to promote tumor metastasis. Histologic investigation revealed that M2‐exos dramatically enhanced the metastatic nodules in the lung, but blocking exosomal ITG αVβ3 inhibited this effect (Figure 5E). These results implied that M2‐like macrophage‐derived exosomal ITG αVβ3 could be transmitted to cancer cells and increase cancer migration and invasion in vivo.

### 2.6 ITG αVβ3 improve tumor metastasis by activating the FAK signaling pathway

**FIGURE 6:**
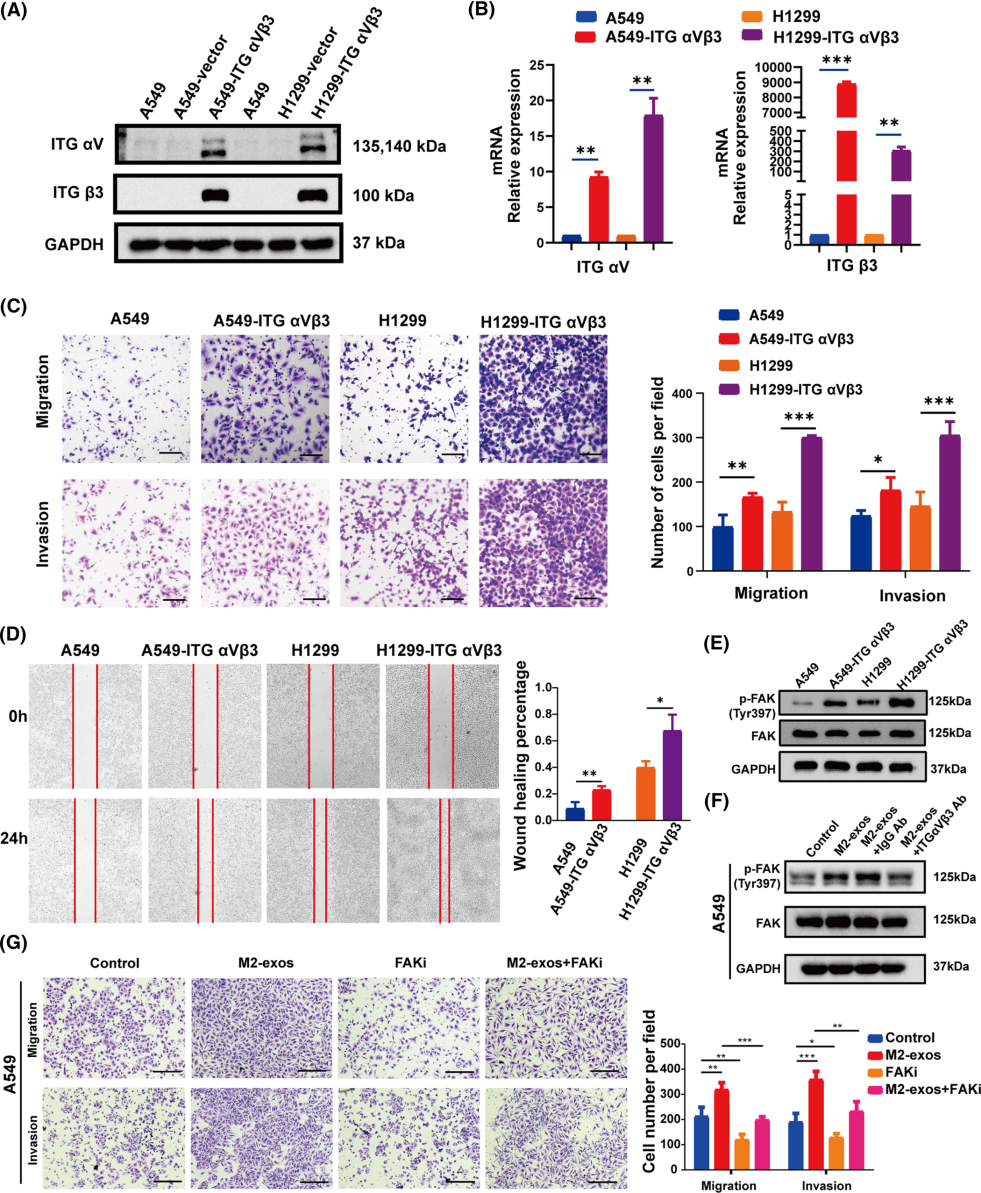
ITG αVβ3 enhances the migration and invasion of non‐small‐cell lung cancer (NSCLC) cells through the focal adhesion kinase (FAK) signaling pathway. (A and B) Western blotting and qPCR were employed to detect the protein and mRNA levels of ITG αV and ITG β3 in A549 and H1299 cells overexpressing ITG αVβ3. (C and D) Transwell and wound healing experiments were used to evaluate the migration and invasion properties of A549 and H1299 cells overexpressing ITG αVβ3. Scale bar, 200 μm. (E) Western blotting were employed to detect the levels of FAK/p‐FAK protein expression in A549 and H1299 cells overexpressing ITG αVβ3. (F) After 24 h of pretreatment with phosphate buffered saline (PBS) (control), M2‐like macrophage‐derived exosomes (M2‐exos), M2‐exos+IgG Ab, or M2‐exos+ ITG αVβ3 Ab, the levels of FAK/p‐FAK protein expression were measured by employing Western blot assays. (G) After 24 h of pretreatment with PBS (control), M2‐exos, FAKi, or M2‐exos+FAKi, cancer cell migration and invasion abilities were measured by employing Transwell assays. Representative images are displayed in the left section, and statistical results are shown in the right section, Scale bar, 150 μm. The previous experiments were repeated at least three times to ensure the accuracy of the data. All data are presented as the means ± SEMs. ITG αVβ3, integrin αVβ3; FAKi, FAK inhibitor, defactinib. *p < 0.05, **p < 0.01, ***p < 0.001

Exosomes have been proven in numerous studies to have a vital function in signal transduction.^39^, ^40^, ^41^ However, the involvement of transportable ITG αVβ3 from M2‐exos in the NSCLC migratory and invasive signaling pathways remains unknown. To further explore the relevant molecular mechanisms, we constructed A549 and H1299 cells overexpressing ITG αVβ3 (Figure 6A,B). We found that A549‐ITG αVβ3 and H1299‐ITG αVβ3 cells had dramatically improved migration and invasion abilities (Figure 6C). We also carried out a wound‐healing assay. The horizontal mobility of A549‐ITGαVβ3 and H1299‐ITG αVβ3 was higher than that of the control group, as expected (Figure 6D). These results further suggested that ITG αVβ3 could be a key effector molecule that promoted tumor metastasis. FAK is a non‐receptor kinase that is primarily responsible for adhesion signaling and cell migration, but it can also promote cell survival in the absence of stress.^42^ Many studies have shown that integrins primarily trigger the FAK signaling pathway, regulating various biological functions.^43^, ^44^ Therefore, we further investigated whether ITG αVβ3 delivered by M2‐exos activated the downstream FAK/p‐FAK signaling pathway in NSCLC cells. We found that both A549‐ITG αVβ3 and H1299‐ITG αVβ3 cells had higher phosphorylated FAK expression than their respective control cells (Figure 6E). Moreover, p‐FAK protein expression was considerably increased in A549 cells after M2‐exo treatment compared to the control group, whereas p‐FAK protein expression was downregulated after preincubation with M2‐exos and ITG αVβ3 blocking antibody (Figure 6F). More importantly, FAK inhibitor treatment significantly offset the increased migration and motility of tumor cells induced by M2‐exos. These results further demonstrated that ITG αVβ3 promoted tumor metastasis by activating the FAK signaling pathway (Figure 6G). Taken together, these results indicated that exosomal ITG αVβ3 derived from M2‐like macrophages is essential for the migration and invasion of NSCLC cells. Mechanistically, intercellularly transferred exosomal ITG αVβ3 primarily activated the FAK signaling pathway to execute biological functions.

### 2.7 Integrin αVβ3 is associated with NSCLC metastasis and poor prognosis in clinic

**FIGURE 7:**
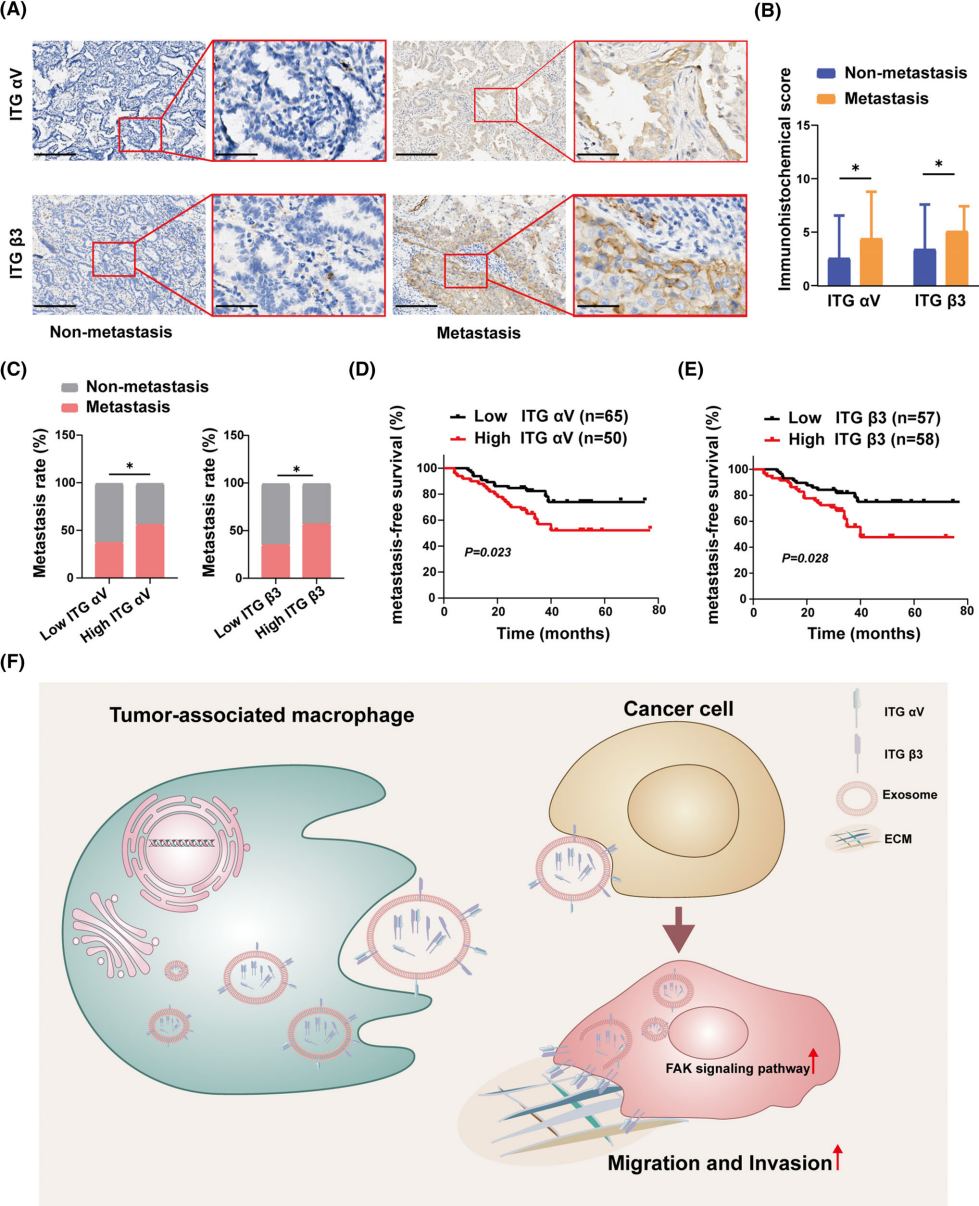
**A high integrin αVβ3 level is associated with non‐small‐cell lung cancer (NSCLC) metastasis and a poor prognosis**. (A and B) Representative images and statistical analysis of ITG αV and ITG β3 expression levels in metastatic and nonmetastatic lung adenocarcinoma specimens. Scale bar, 200 and 50 μm. (C) Comparison of metastatic rates among lung adenocarcinomas with high and low levels of ITG αV and β3 expression. (D and E) Relationship between ITG αV and β3 expression levels and metastasis‐free survival. (F) Schematic diagram of exosomal integrin αVβ3 from M2‐like macrophages facilitating tumor cell metastasis via the focal adhesion kinase (FAK) signaling pathway

We revealed that M2‐like macrophage‐derived exosomes mediate ITG αVβ3 transmission to increase NSCLC migration and invasion in vitro *and* in vivo. To verify the reliability of this conclusion in real data, we collected specimens of 126 cases of LUAD specimens, including metastatic cases (*n* = 59) and nonmetastatic cases (*n* = 67) (Table 1). Immunohistochemistry was used to determine the levels of ITG αV and β3 expression in lung cancer specimens. We discovered that LUAD s with metastasis had greater levels of ITG αV and β3 expression than those without metastasis (Figure 7A,B). Then, the specimens were divided into high or low ITG αV groups and high or low ITG β3 groups based on immunohistochemical scores. We found that the rate of metastasis was higher in the high ITG αV and high ITG β3 groups than in the correspondingly low score groups (Figure 7C). This suggested that high expression of ITG αV and ITG β3 in tumor cells could indicate a poor prognosis. Therefore, we analyzed metastasis‐free survival in each group. Compared with the low expression group, the high expression group had worse metastasis‐free survival (Figure 7D,E). Overall, this clinical evidence suggested that high expression of ITG αV and ITG β3 was associated with a poor prognosis.

**TABLE 1:** Correlation between metastasis and clinical pathology characteristics in lung cancer

		Metastasis			
Variable	No.	Nonmeta	Meta	*X* ^2^	*p* Value
Age					
<60	59	28 (47.5%)	31 (52.5%)	1.456	0.228
>60	67	39 (58.2%)	28 (41.8%)		
Gender					
Female	57	31 (54.4%)	26 (45.6%)	0.061	0.804
Male	69	36 (52.2%)	33 (47.8%)		
Smoking					
Nonsmoking	79	42 (53.2%)	37 (46.8%)	0.033	0.856
Smoking	47	25 (53.2%)	22 (46.8%)		
ITG β3					
Low expression	62	40 (64.5%)	22 (35.5%)	6.306	0.012
High expression	64	27 (42.2%)	37 (57.8%)		
ITG αV					
Low expression	66	41 (62.1%)	25 (37.9%)	4.455	0.035
High expression	60	26 (43.3%)	34 (56.7%)		

*Note*: Nonmeta: the nonmetastatic group, meta: the metastatic group.

## 3 DISCUSSION AND CONCLUSION

Currently, the vast majority of cancer‐related deaths (approximately 90%) are caused by metastatic disease.^45^ Tumor metastasis is a complex biological process involving multiple cascade steps, and there are still many unexplained mechanisms.^46^ Some studies have shown that the TME enriches a variety of immunosuppressive cells, which have a vital role in mediating the invasiveness of primary tumors and the ability to metastasize to distant sites through direct contact with cancer cells or paracrine pathways.^47^, ^48^ Macrophages are the main tumor‐infiltrating leukocytes in almost all cancers. They can be “domesticated” by tumor cells to polarize toward M2‐like macrophage phenotype, therefore supporting tumor progression.^48^ Many studies have reported that the invasion and metastasis of NSCLC are closely related to its microenvironment.^49^ However, the link between the metastasis of NSCLC and macrophages and the underlying molecular mechanism have not yet been clarified.

It has been reported that TAMs in the TME can be differentiated from bone marrow‐derived macrophages or tissue‐colonized macrophages.^48^ In NSCLC, tumor‐promoting TAMs are mainly derived from bone marrow macrophages.^50^ Therefore, in this study, we simulated the physiological effects of TAMs in vivo by inducing the polarization of acute leukemia monocytes THP‐1 to M2‐like macrophages in vitro. A study performed by Lee et al. showed that TAMs in metastatic tumors are mainly M2 macrophage phenotypes.^51^ Many studies have reported that M2 macrophages released a wide range of chemokines, cytokines to enhance tumor invasion and metastasis.^52^ Nevertheless, the molecular mechanism by which they interact with tumor cells is unknown.

An increasing number of studies have shown that exosomes produced from tumor‐associated stromal cells have a significant role in mediating intercellular communication.^33^ For example, exosomes derived from tumor‐associated fibroblasts promoted the metastasis of colorectal cancer cells and chemotherapy resistance.^37^ Similarly, M2‐exos were confirmed to increase the metastasis of colorectal cancer cells.^33^ In addition, Wu et al. found that M2 macrophage‐derived exosomes delivered integrin α_M_β2 to hepatocellular carcinoma cells and activated the expression of MMP9, thereby promoting the invasion and metastasis of cancer cells.^53^

In addition to determining the colonization of metastatic sites and promoting the nonanchoring survival of circulating tumor cells, integrins, as the primary cell adhesion receptors, can also interact with multifunctional cell surface molecules (including channels, receptors, and secreted proteins) to mediate signaling and cell migration and invasion. In conclusion, integrins play a multifaceted and important role in almost every step of cancer progression from primary tumor to metastasis.^54^ Consistently, our research has shown that exosomes secreted by M2‐like macrophages possess the ability to enhance the metastasis of NSCLC cells in vivo and in vitro. Exosomes are composed of a range of biologically active components, including proteins, RNA, DNA and lipids.^28^, ^55^ Importantly, exosomal contents derived from different cell types are unique. Our study found that M2‐exos showed a significant enrichment of ITG αVβ3. ITG αVβ3 is a marker of tumor angiogenesis, and its expression on tumor cells is related to cancer progression, drug resistance, and EMT.^56^ In addition, Wettersten et al. showed that ITG αVβ3 has a significant positive correlation with TAM markers in various cancers.^57^ Additionally, the enrichment of ITG αVβ3 was found in prostate cancer cell‐derived exosomes, which could promote the migration phenotype of non‐tumorigenic cells through intercellular delivery of exosomes.^58^ In this study, we demonstrated that M2‐exo‐mediated invasion and metastasis of NSCLC cells are dependent on ITG αVβ3. Exogenous blockade of ITG αVβ3 derived from M2‐exos prevented the migration and invasion of NSCLC induced by M2‐exos.

Studies thus far have shown that exosomes are important mediators of intercellular signal transduction. However, the mechanism by which ITG αVβ3 transferred by M2‐exos drives the migration and invasion of NSCLC is still unclear. FAK is a major regulator of growth factor receptor and integrin‐mediated signals, which controls the basic processes of normal cells and cancer cells through kinase activity and scaffold function.^59^ FAK activity and expression are upregulated in many cancers and are usually relevant to poor clinical outcomes, implying that FAK could be used to predict tumor progression.^59^

In this study, we found that M2‐exos ITG αVβ3 mainly facilitated the invasion and metastasis of NSCLC by activating FAK signal transduction. Our research showed that M2‐exos were rich in ITG αVβ3, which could be directly transferred to NSCLC cells, resulting in accelerated migration and invasion in NSCLC. ITG αVβ3 facilitated NSCLC invasion and metastasis by increasing the phosphorylation of FAK. Blocking exosomal ITG αVβ3 weakened the potential of M2‐exos to increase NSCLC cell migration and invasion (Figure 7F). Therefore, our study supported ITG αVβ3 as a biomarker of TAM activation in NSCLC. In addition, blocking the ITG αVβ3‐FAK signal transduction pathway may be a promising treatment to control the metastasis of NSCLC. However, more research is still needed to further clarify its potential mechanism.

## 4 MATERIALS AND METHODS

### 4.1 Cell culture

The human NSCLC cell Lines A549 and H1299 were cultured in DMEM (Gibco), and the human acute monocytic leukemia cell line THP‐1 was cultured in RIPA 1640 medium (Gibco). Both media contained 1% penicillin–streptomycin (Gibco) and 10% fetal bovine serum (FBS, Gibco). For macrophage polarization, THP‐1 cells were treated with 100 nM phorbol‐12‐myristate‐13 acetate (PMA, Sigma‒Aldrich, P1585) for 48 h, after which the medium was discarded and the cells were washed twice with pre‐warmed PBS. The PMA‐differentiated THP‐1 macrophages were then cultured for another 24 h in the RPMI 1640 complete medium (without PMA) to obtain the resting state of macrophages (M0). For M1‐ or M2‐like macrophages polarization, M0 macrophages were cultured for 48 h in the medium supplemented with 100 ng/ml lipopolysaccharide (LPS, Sigma‒Aldrich, L2630) and 20 ng/ml IFN‐γ (PeproTech, 300–02) or 20 ng/ml IL‐4 (PeproTech, 200–04) and IL‐13 (PeproTech, 200–13), respectively. All cells were cultured at 37°C in a 5% CO_2_ atmosphere.

### 4.2 Exosome isolation

THP‐1‐differentiated M2‐like macrophages were cultivated for 24 h in an FBS‐free RIPA 1640 medium, which was collected and exosomes were extracted by differential ultracentrifugation as described previously.^60^ Briefly, to remove cells and debris, the conditioned media was centrifuged at 300 × *g* for 5 min and 2000 × *g* for 15 min. Then, the supernatant was harvested and centrifuged at 15,000 × *g* for 30 min at 4°C to eliminate large EVs. Exosomes were isolated by centrifugation (Beckman Coulter Avanti J30I) at 100,000 × *g* for 90 min. Finally, the isolated exosomes were resuspended in 200 μl PBS and used immediately or stored at −80°C.

### 4.3 Exosome identification

TSG101 (Affinity, DF8427), CD63 (Affinity, DF2305), and ALIX (Affinity, DF9027) were utilized as positive controls in Western blot analysis, whereas Calnexin (Affinity, AF5362), an endoplasmic reticulum protein, was used as a negative control. The NanoSight NS300 system (NanoSight Technology, Malvern, UK) was employed to directly monitor the number and size distribution of exosomes.

### 4.4 Exosome uptake assays

The extracted exosomes were treated with PKH26 Fluorescent Cell Linker Kits (Sigma‒Aldrich, PKH26GL) according to the manufacturer's protocol to visualize exosome internalization. Next, the tagged exosomes were cultured with H1299 cells for 6 h. The cells were fixed in 4% paraformaldehyde for 30 min before being stained using Abcam's CytoPainter Phalloidin‐iFluor 488 Reagent for 30 min. The nuclei were then stained with Hoechst 33342 (Cell Signaling Technology, Danvers, MA) for 10 min. A confocal microscope was used to examine how H1299 cells took up exosomes.

### 4.5 Transwell assay

For cell migration experiments, 2 × 10^4^ NSCLC cells were resuspended in 200 μl of FBS‐free media and seeded into 24‐well Transwell cell culture chambers (8 μm pore size, BD), and 650 μl of medium containing 10% FBS was added to the lower chamber. For cell invasion assays, 4 × 10^4^ NSCLC cells were resuspended in 200 μl of FBS‐free media and planted into the upper inserts with pre‐coated Matrigel, and 650 μl of media containing 10% FBS was added to the lower chamber. For NSCLC cell and M2 macrophage indirect cocultured assays, 2 × 10^4^ THP‐1 cells were seeded into the lower chamber and induced to polarize toward M2 macrophages according to the previous protocols. Then, NSCLC cells were harvested and suspended in 200 μl of FBS‐free DMEM before being transferred to the upper compartment. The cells in the upper chamber were wiped out after 24 h, and the cells in the lower chamber were fixed with 4% paraformaldehyde and stained with 0.5% crystal violet. To identify immune molecules in M2‐exos that induce NSCLC cell migration and invasion, A549 and H1299 cells were cocultured with M2‐exos, M2‐exos+anti‐IgG blocking antibody (M2‐exos+IgG Ab), and M2‐exos+anti‐ITG αVβ3 blocking antibody (M2‐exos+ ITG αVβ3 Ab, BioLegend, 327902) for 24 h. Control group NSCLC cells were incubated with PBS. The NSCLC cell migration and invasion results were photographed and counted. At least five random microscopic fields were taken, and the cells were counted. All experiments were performed in triplicate.

### 4.6 Flow cytometry staining and analysis

Flow cytometric assays were used to evaluate the expression of CD206 and HLA‐DR as previously described.^61^ Briefly, 5×10^5^ M1 and M2 macrophages were harvested and stained with PE‐CD206 antibody (BioLegend, 321105) or FITC‐HLA‐DRα antibody (BioLegend, 374208) for 15–20 min and subsequently analyzed using flow cytometry. Flow cytometry data were analyzed by FlowJo (Treestar) software.

### 4.7 Western blot analysis

Briefly, whole‐cell lysates were electrophoresed in an 8% SDS–PAGE gel and then transferred to 0.22 m PVDF membranes (Millipore) after being lysed in an RIPA buffer with protease inhibitors. The membranes were blocked for 1 h at 37°C in TBST with 5% skimmed milk powder before being probed with the specific antibody (ITG β1, 1:1000, Cell Signaling Technology, 9699S; ITG β3, 1:1000, Cell Signaling Technology, 13166T; ITG β4, 1:1000, Cell Signaling Technology, 4707S; ITG β5, 1:1000, Cell Signaling Technology, 4708S; ITG α2, 1:1000, Cell Signaling Technology, 13807S; ITG αV, 1:1000, Cell Signaling Technology, 4711S; ITG α6, 1:1000, Cell Signaling Technology, 3750S; GAPDH, 1:5000, Proteintech, 6004‐1‐1g; p‐FAK(Tyr397), 1:1000, Cell Signaling Technology, 8556S; FAK, 1:1000, Cell Signaling Technology, 71433S) overnight at 4°C. Then, the membranes were incubated with secondary antibody (1:5000) for 1 h at 37°C. The protein bands were identified using an ECL detection system (Bio‐Rad, USA).

### 4.8 Reverse transcription and quantitative real‐time PCR

Total cellular RNA was extracted using a TRIzol reagent (Invitrogen,), and 1 μg total RNA was reverse transcribed into first‐strand complementary DNA (cDNA) using a cDNA Synthesis Kit (EZBioscience) according to protocols. Afterwards, cDNA was used to measure the relative gene expression level using real‐time PCR. The expression of target genes was normalized to GAPDH levels in the samples in triplicate. The 2^−ΔΔ^ *^CT^* method was used to calculate the relative variation in gene expression. Additional file: Table S1 contains a list of primers.

### 4.9 Animals

Male Balb/c nude mice aged 4–6 weeks were purchased from Guangdong Medical Laboratory Animal Center in China. To establish a human NSCLC lung metastasis model in nude mice, 3 × 10^6^ A549^luc^ cells were resuspended in 200 μl FBS‐free DMEM and injected intravenously into Balb/c nude mice. Nude mice were randomly divided into four groups based on body weight. To study the blockade effects of ITG αVβ3, 10 μg M2‐exos, M2‐exos+ITG αVβ3 Ab, and M2‐exos+IgG Ab were administered to Balb/c nude mice every 4 days. A similar volume of PBS was injected into the control group. Mice were sacrificed after 50 days, and the lungs were assessed for metastatic lesions by comparing biofluorescence signal intensities. Tissue morphology was identified by hematoxylin and eosin staining.

### 4.10 Construction and transfection of ITG αVβ3 shRNA and overexpression plasmids

As previously described, reliable knockdown and overexpression cell lines were established.^62^ Lentiviral vectors were used to create ITG αVβ3 shRNA and overexpression plasmid. The generated plasmid was co‐transfected into 293T cells for 48 h with the viral packaging plasmids psPAX2 and pMD2.G. Lentiviral supernatants were harvested and filtered through 0.45 μm filter before being cultivated for 24 h with H1299 cells. Puromycin selection (2 g/ml) was applied to the cells. Additional file: Table S2 shows the targeting sequences for specific genes. Table S3 shows the primers for overexpressed genes.

### 4.11 Immunohistochemistry

Clinical tumor specimens were gathered at the Sun Yat‐sen University Cancer Center in Guangzhou, China, who had been diagnosed with NSCLC. For patient specimens, all patients gave their agreement and enrolled in IRB‐approved protocols at Sun Yat‐sen University Cancer Center, which allowed the collection and analysis of clinical data and archival and paraffin specimens in compliance with ethical principles (Ethics Document No. SL‐B2022‐139‐01). Tumor specimens were formalin‐fixed and paraffin‐embedded, as is standard laboratory pathology technique, and stored at the Sun Yat‐sen University Cancer Center's pathology department. The paraffin slices from patients' tissues were treated with primary antihuman antibodies at various dilutions (ITG αV, 1:400, Beyotime, AF7308; ITG β3, 1:200, Beyotime, AF1444) overnight at 4°C. They were then treated for 60 min at room temperature with the second antibody. The staining was identified by using a DAB Kit (Zisbio) as directed by the manufacturer. Hematoxylin staining was measured using at least five randomly selected fields of 200x or 400x after slides were stained for 6 min. Two pathologists independently evaluated the protein expression.

### 4.12 Statistical analysis

Unless otherwise specified, the results are presented as the means ± SEMs and were analyzed using one‐way ANOVA or Student's *t* test. Experiments were repeated at least three times to ensure the accuracy of the data. The statistical significance level was set at *p* < 0.05. SPSS 22.0 or GraphPad Prism 7 was used for all statistical analyses.

## AUTHOR CONTRIBUTIONS

Liwu Fu and Jianye Zhang conceived of the study. Liwu Fu and Fang Wang designed it. Lamei Huang, Fang Wang, Xueping Wang, Chaoyue Su, Shaocong Wu, Chuan Yang, and Min Luo carried out the experiments. Lamei Huang and Fang Wang analyzed and interpreted the data. Lamei Huang and Jianye Zhang drafted the manuscript with comments from all authors. Jianye Zhang and Liwu Fu reviewed the manuscript. Lamei Huang and Fang Wang contributed equally. All authors read and approved the final manuscript.

## CONFLICTS OF INTEREST

The authors have no relevant or potential conflicts of interest to declare.

## ETHICS STATEMENT

All experiments involving animals were conducted according to the ethical policies and procedures approved by the ethics committee of Sun Yat‐sen University Cancer Center (Approval no. L102012019120R). . For patient specimens, all patients gave their agreement and enrolled in IRB‐approved protocols at Sun Yat‐sen University Cancer Center, which allowed the collection and analysis of clinical data and archival and paraffin specimens in compliance with ethical principles (Ethics Document NO. SL‐B2022‐139‐01).

## Supporting information

Supporting Information

## Data Availability

All data generated or analyzed during this study are included in this published article and its Supporting Information files.
