# Supplementary material for: M2‐like macrophage‐derived exosomes facilitate metastasis in non‐small‐cell lung cancer by delivering integrin αVβ3
Source: MedComm (2020). 2022 Dec 23;4(1):e191. doi: 10.1002/mco2.191 (PMC9789322; doi:10.1002/mco2.191)
Supplement: Supplementary file 1 — Supporting Information [file MCO2-4-e191-s001.docx]

**M2-like macrophage**-**derived** **exosome****s facilitate metastasis in non-small-cell lung cancer by delivering integrin αⅤβ3**

Lamei Huang^1#^, Fang Wang^1#^, Xueping Wang^1^, Chaoyue Su^2^, Shaocong Wu^1^, Chuan Yang^1^, Min Luo^1^, Jianye Zhang^2^*, Liwu Fu^1^*

^1^ State Key Laboratory of Oncology in South China; Collaborative Innovation Center for Cancer Medicine; Guangdong Esophageal Cancer Institute; Sun Yat-Sen University Cancer Center, Guangzhou, 510060, P. R. China

^2^Guangzhou Municipal and Guangdong Provincial Key Laboratory of Molecular Target & Clinical Pharmacology, the NMPA and State Key Laboratory of Respiratory Disease, School of Pharmaceutical Sciences and the Fifth Affiliated Hospital, Guangzhou Medical University, Guangzhou 511436, China

^#^Contributed equally.

**Running title**: Macrophage-derived exosomes facilitate metastasis

***Correspondence to:** Jianye Zhang, School of Pharmaceutical Sciences & the Fifth Affiliated Hospital, Guangzhou Medical University, Guangzhou, P. R. China. E-mail: [jianyez@gzhmu.edu.cn](mailto:jianyez@gzhmu.edu.cn). Liwu Fu, Sun Yat-sen University Cancer Center, Guangzhou, 510060, China; Tel.:+86-20-873-431-63; Fax: +86-20-873-431-70; E-mail: [fulw@mail.sysu.edu.cn](mailto:fulw@mail.sysu.edu.cn)

| **Primers** | **Sequences (5'-3')** |
| --- | --- |
| CD206 Forward | CTGAATTGTACTGGTCTGTCCT |
| CD206 Reverse | GCTTAGATGTGGTGCTGTGG |
| CD163 Forward | CCGGGAGATGAATTCTTGCCT |
| CD163 Reverse | AGACACAGAAATTAGTTCAGCAGCA |
| ARG-1 Forward | TCATCTGGGTGGATGCTCACAC |
| ARG-1 Reverse | GAGAATCCTGGCACATCGGGAA |
| HLA-DRα Forward | AGCTGTGGACAAAGCCAACCTG |
| HLA-DRα Reverse | CTCTCAGTTCCACAGGGCTGTT |
| TNF-α Forward | CTGGCCCAGGCAGTCAGATCATC |
| TNF-α Reverse | CTTGAGGGTTTGCTACAACATG |
| iNOS Forward | TTCAGTATCACAACCTCAGCAAG |
| iNOS Reverse | TGGACCTGCAAGTTAAAATCCC |
| Integrin αV Forward | AGGAGAAGGTGCCTACGAAGGT |
| Integrin αV Reverse | GCACAGGAAAGTCTTGCTAAGGC |
| Integrin β3 Forward | CATGGATTCCAGCAATGTCCTCC |
| Integrin β3 Reverse | TTGAGGCAGGTGGCATTGAAGG |
| GAPDH Forward | GAAGGTGAAGGTCGGAGTCAACG |
| GAPDH Reverse | TGCCATGGGTGGAATCATATTGG |

**Table S1. qPCR primers for screening and validation**

**Table S2. shRNAs for ITG αV and β3**

| **Gene** | **Sequences (5'-3')** |
| --- | --- |
| Integrin αV-sh 1 | GTGAGGTCGAAACAGGATAAA |
| Integrin αV-sh 2 | CGACAGGCTCACATTCTACTT |
| Integrin β3-sh 1 | CCACGTCTACCTTCACCAATA |
| Integrin β3-sh 2 | CCTTAGCCTTTGTCCCAGAAT |

**Table S3. overexpression for ITG αV and β3**

| **Gene** | **Primer (5'-3')** |
| --- | --- |
| Integrin αV-F1 | GCACTTCGGCGATGGCTTTT |
| Integrin αV-R1 | CAGGTTAGTTTCTGAGTTTCCTTCACC |
| Integrin β3-F1 | AGGCGGACGAGATGCGAG |
| Integrin β3-R1 | TTAAGTGCCCCGGTACGTG |

**
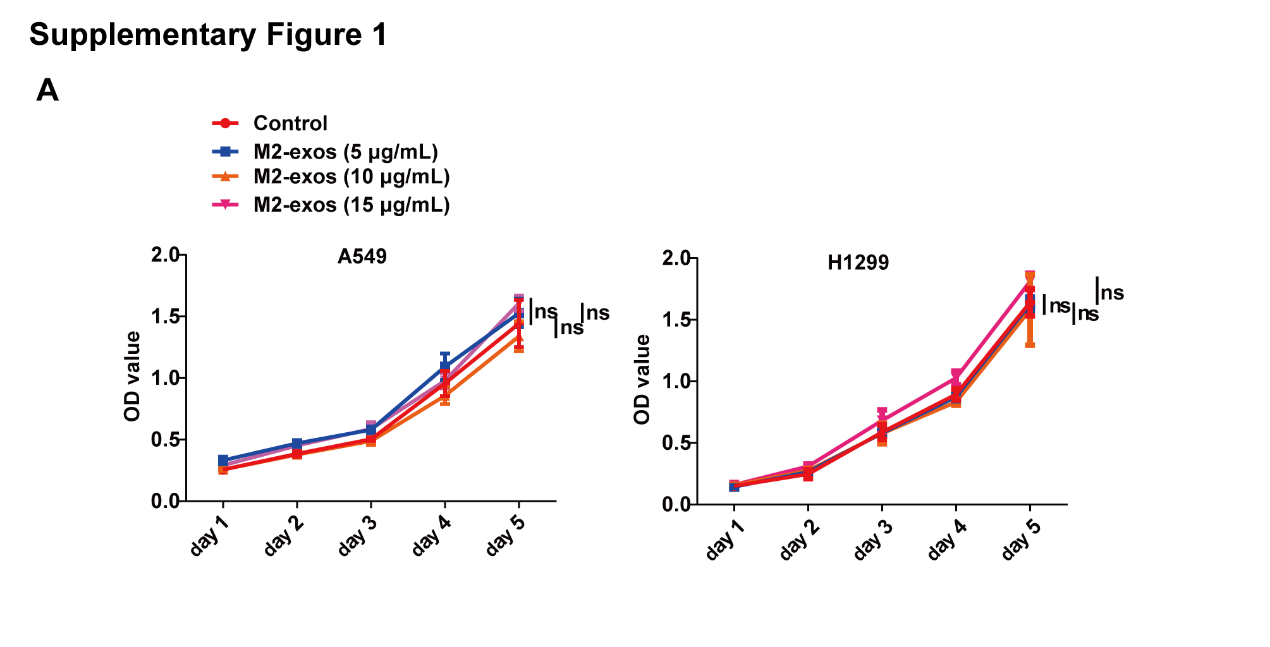
**

Figure S1. MTT tests were used to determine the growth rate of A549 and H1299 cells after treatment using different doses of M2-exos.
